# Case Report: Infant with vaccine-associated paralytic poliomyelitis unveils global disparities in care for inborn errors of immunity

**DOI:** 10.3389/fimmu.2026.1751423

**Published:** 2026-02-06

**Authors:** Chee Mun Chan, Elisabeth Sue Shuen Fong, Daryl Yuan Chong Yeo, Elizabeth Y. Ang, Isabella Ming Zhen Liu, Hian Tat Ong, Si Min Chan, Hui-Lin Chin, Frances Shi Hui Yeap, Chee Kwang Kee, Teresa Shu Zhen Tan, Nicholas Beng Hui Ng, Youjia Zhong, Jeremy Bingyuan Lin, Lydia Su Yin Wong

**Affiliations:** 1 Khoo Teck Puat National University Children’s Medical Institute, National University Hospital, Singapore, Singapore; 2 Department of Paediatrics, Yong Loo Lin School of Medicine, National University of Singapore, Singapore, Singapore; 3 Department of Diagnostic Imaging, National University Hospital, Singapore, Singapore; 4 Infectious Diseases Translational Research Programme, Yong Loo Lin School of Medicine, National University of Singapore, Singapore, Singapore; 5 Programme in Emerging Infectious Diseases, Duke-NUS Medical School, Singapore, Singapore

**Keywords:** healthcare disparity, immunodeficiency, Omenn Syndrome, severe combined immunodeficiency, vaccine-associated paralytic poliomyelitis

## Abstract

Severe combined immunodeficiency (SCID) is a life-threatening inborn error of immunity (IEI) that is potentially curable when diagnosed early but is associated with high morbidity and mortality when recognition is delayed. In settings without universal newborn screening (NBS), and where vaccination programs rely heavily on live vaccines, affected infants are at risk of preventable complications.

We report an Indonesian girl, the first child of non-consanguineous parents, who at the age of three months, developed a generalized maculopapular rash, followed by febrile encephalopathy with acute flaccid paralysis and hepatomegaly at four months of age. Investigations revealed pan-hypogammaglobulinemia (IgG <1.09 g/L, IgA <0.05 g/L, IgM 0.06 g/L) and marked eosinophilia (7554 cells/uL). On transfer to Singapore, detailed immunophenotyping revealed T cell lymphocytopenia with the vast majority being 99.3% CD45RO+ on CD3+CD4+ cells, and absent B cells. The diagnosis of SCID with Omenn syndrome was made, with genetic analysis revealing compound heterozygosity for pathogenic *RAG1* variants. As the child received oral polio vaccine (OPV) at day 8 of life, vaccine-associated paralytic poliomyelitis (VAPP) was suspected, which was confirmed with positive enterovirus PCR in the cerebral spinal fluid; Sabin-like poliovirus serotype 1 was isolated from stool. Hematopoietic stem cell transplantation (HSCT) was discussed but the family opted for supportive palliative care. The child died of a febrile illness at 8 months of age.

Although IEI was initially suspected, the use of limited flow cytometric diagnostic evaluation delayed the definitive diagnosis in the child. The lack of NBS for SCID, together with the continued usage of OPV in the routine childhood vaccination program in most lower to middle income countries is the perfect storm for VAPP in children born with IEIs in these settings. This case highlights that 1) there is an urgent need to strengthen diagnostic capabilities in resource-limited settings, 2) the transition from OPV to inactivated polio vaccine (IPV) is a public health priority, and 3) there are significant barriers to the implementation of SCID NBS in Southeast Asia. Addressing these systemic gaps is critical to improve survival outcomes for children with severe but treatable IEIs.

## 1 Introduction

Severe combined immunodeficiency (SCID), an inborn error of immunity (IEI), is a pediatric emergency marked by profound T-cell impairment with or without B- and NK-cell defects, if untreated, it is uniformly fatal in early life. Although global incidence is often quoted at 1:50,000–1:100,000 live births, the true burden in Southeast Asia is uncertain due to under-recognition and heterogeneous diagnostic capacity, including limited flow cytometry panels and constrained access to molecular tests (1). Regional analyses using the iPOPI Primary Immunodeficiency (PID) Life Index highlight persistent care gaps such as availability of diagnostic tools and access to clinical immunology specialist care (2).

Newborn screening (NBS) using T-cell receptor excision circles (TREC) from a single dried blood spot sample is a validated and cost-effective strategy. It enables pre-symptomatic identification of SCID and timely access to definitive therapy such as hematopoietic stem cell transplantation (HSCT), when children are still infection-free (3). Population-based programs have demonstrated high sensitivity for typical SCID, with marked reductions in infection-related morbidity and mortality when screening is implemented prior to symptom onset (3, 4). To broaden detection beyond isolated T-cell lymphopenia, combined TREC and kappa-deleting recombination excision circle (KREC) assays have been evaluated. In a retrospective Bulgarian study analyzing 2,228 dried blood spot samples, TREC/KREC screening identified 100% of SCID cases at birth, with TREC correlating with CD4^+^ and total T-cell counts and KREC reliably predicting B-cell lymphopenia (5). More recently, second-tier next-generation sequencing (NGS) following an abnormal TREC result has enabled rapid molecular diagnosis, informed targeted management and follow-up, and improved detection of atypical or leaky SCID and other combined immunodeficiencies (6). The advent of NBS has been a breakthrough for the management of SCID.

Despite clear clinical benefit, implementation of NBS for SCID remains uneven across Southeast Asia due to economic and health-system constraints (1). Key barriers include the costs of reagents and specialized equipment, limited laboratory infrastructure and trained personnel, confirmatory testing, and referral pathways (1, 2, 4). Broader system-level factors, such as fragmented care networks, unequal access to immunology and transplant services, and variability in national policy adoption, further contribute to heterogeneous screening coverage across the region (1). These challenges highlight that successful SCID NBS requires not only technical feasibility but also coordinated investment in laboratory capacity and health-system integration tailored to local contexts.

While HSCT is curative for SCID, outcomes are strongly dependent on age and clinical status at transplantation. Large cohort studies and global reviews consistently demonstrate overall survival rates exceeding 90% when HSCT is performed within the first 3–4 months of life, particularly in infection-free infants identified through NBS, whereas delayed transplantation and active infections are associated with poor outcomes (7–9). In many low- and middle-income settings, however, delayed recognition intersects with continued use of live vaccines very early in the routine childhood vaccination schedule. Specifically, the use of oral poliovirus vaccine (OPV) exposes undiagnosed SCID infants to vaccine-associated paralytic poliomyelitis (VAPP) (10, 11). Because there is no effective antiviral treatment for VAPP, infection is often progressive and irreversible. Moreover, chronic poliovirus infection in immunocompromised hosts generates vaccine-derived polioviruses (VDPVs), that potentially hamper global polio eradication efforts (10).

In this report, we describe an Indonesian infant with *RAG1*-related Omenn syndrome presenting with OPV-associated polioencephalomyelitis. Our case illustrates how global disparities in diagnostic tools, SCID NBS implementation and polio vaccination programs can culminate into preventable mortality in resource-limited regions (12–14).

## 2 Case description

**Figure 1:**
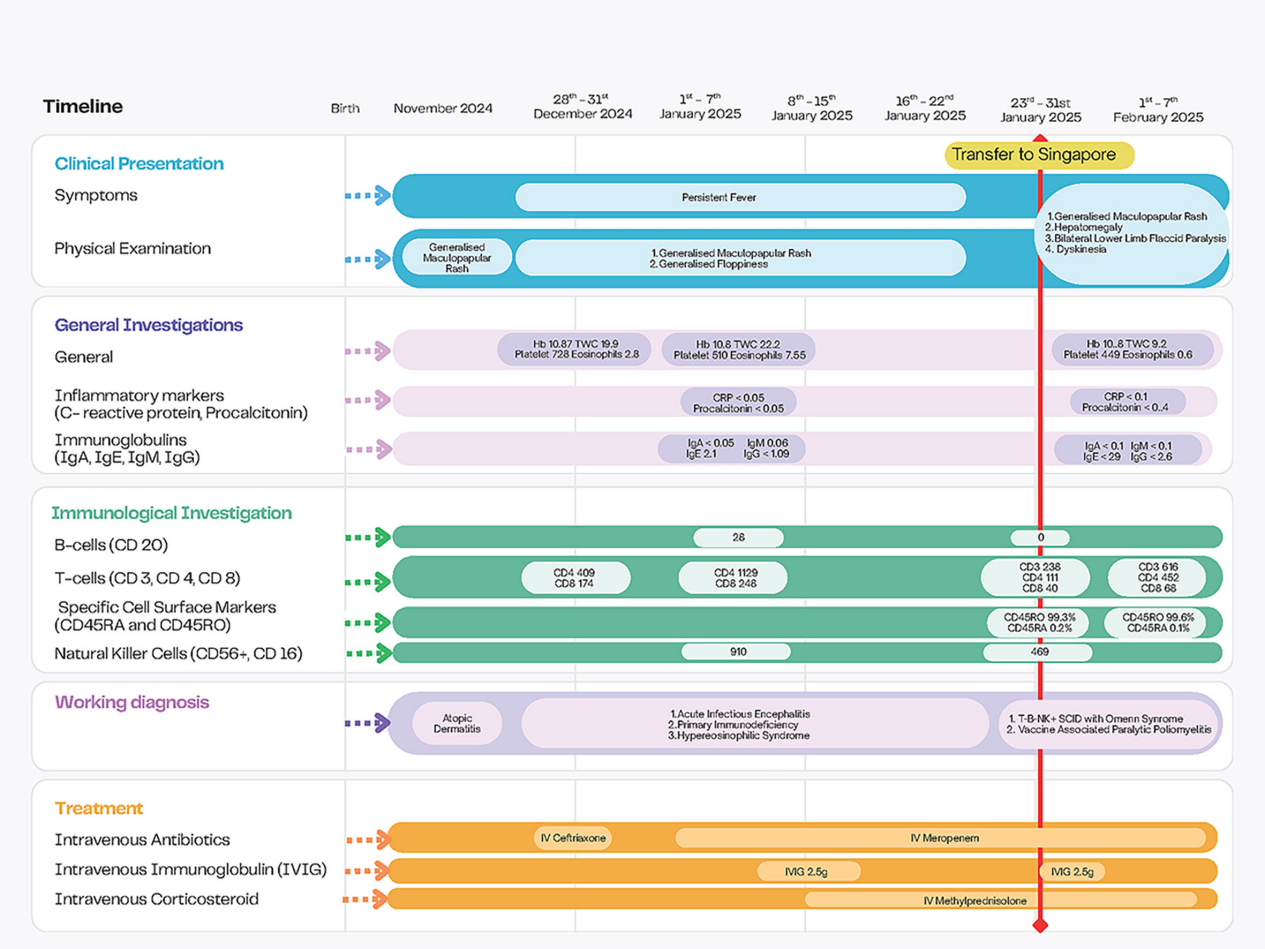
Timeline highlighting significant events including clinical features, significant investigation results, working diagnoses, and treatment. Normal reference range of immunoglobulins: Immunoglobulin A (IgA), 0.0-0.3g/L; Immunoglobulin E (IgE), ≤ 440 IU/ml; Immunoglobulin M (IgM), 0.2-0.9 g/L; Immunoglobulin G (IgG), 1.1-7.0 g/L. Normal reference range of lymphocyte subsets: CD3+:2765-3516/ul; CD4+: 1687-2424/uL; CD8+: 751-1137/uL; CD20+: 989-1696/uL; CD56+CD16+: 302-846/uL [13]. Other abbreviations (units): Hemoglobin (Hb), g/dL; Total White Cells (TWC), x10^9^/L; Platelets, x1^9^/L; C-reactive Protein (CRP), mg/L; Procalcitonin, ng/mL. Eosinophils, x10^9^/L.

**Figure 2:**
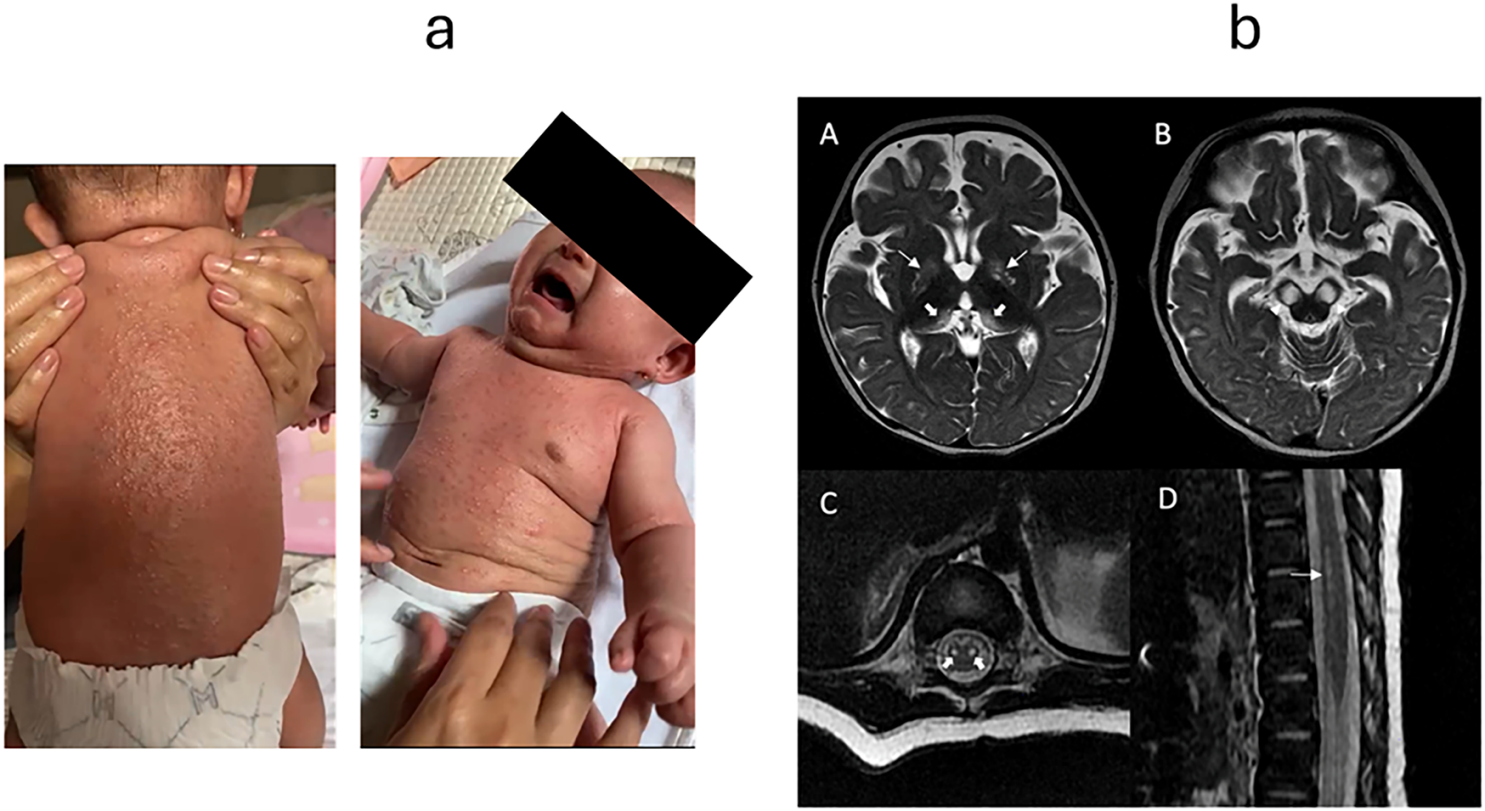
**(a)** Generalized maculopapular rash before treatment in Indonesia. **(b)** Magnetic resonance imaging (MRI) brain and spine Selected axial T2-weighted MRI brain images showing symmetrical T2-weighted hyperintense signal involving the **(A)** globus pallidi (thin arrows), posteromedial thalami (thick arrows), and **(B)** substantia nigra. Selected axial **(C)** and sagittal **(D)** T2-weighted MRI whole spine images showing T2-weighted hyperintense signal involving the anterior horns of spinal cord from T10-T12 level (thick and thin arrows in C and D, respectively).

A 5-month-old Indonesian girl was transferred to Singapore with a one-month history of febrile encephalopathy, lower limb paralysis, and dyskinesia (**Figure 1**) (15). She was the first child of non-consanguineous parents, conceived via *in-vitro* fertilization (IVF), and delivered at 42 weeks’ gestation by elective caesarean section with birth weight of 2.8 kilograms. The antenatal course was unremarkable, and there was no known family history suggestive of immune deficiency or dysregulation. She was well at birth and until three months of age, when she developed a generalized maculopapular rash (**Figure 2a**). The rash was initially treated as atopic dermatitis, but it was unresponsive to topical corticosteroids. At four months of age, she developed five days of fever accompanied by acute neurological symptoms, including drowsiness, a tense anterior fontanelle, abnormal involuntary tongue movements, and flaccid paralysis of bilateral lower limbs. Blood investigations in Indonesia revealed normal inflammatory markers and negative bacterial cultures. Additionally, she was found to have pan-hypogammaglobulinemia (IgG <1.09 g/L, IgA <0.05 g/L, IgM 0.06 g/L), B cell lymphocytopenia (CD20+ cells = 28 cells/uL) and marked eosinophilia (7554 cells/uL). T cell counts were low initially (CD4+ cells = 409/uL, CD8+ cells = 174/uL) but became normal when retested 5 days later (**Table 1**). She was given the working diagnosis of “acute infectious encephalitis, primary immunodeficiency, and hypereosinophilic syndrome”, and treated with broad-spectrum antibiotics, immunoglobulin replacement and multiple doses of intravenous methylprednisolone (~2mg/kg/day). With this treatment, there was some clinical improvement in both consciousness and rash.

**Table 1:** Investigations done in Singapore.

Investigation	Results
Blood investigations	
HIV Ab-Ag	Non-reactive
Serum CMV DNA PCR	Not detected
Serum EBV DNA PCR	Not detected
Hepatitis B Surface antigen	Non-reactive
Hepatitis B Surface antibody (>10 IU/L considered Hep B immune)	86 IU/L
Hepatitis C antibody	Non-reactive
Aspergillus galactomannan (<0.5)	0.07 (negative)
Blood culture	No growth after 5 days
Blood fungal culture	No growth
AFB culture	No growth
Cerebral spinal fluid investigations	
CSF protein (0.2 - 0.8 g/L)	0.34 g/L
CSF glucose (3.3 - 4.5 mmol/L)	2.7 mmol/L
CSF cultures	No bacterial growth. Gram smear 1+ (occasional WBC), no organisms seen
Fungal smear	No fungal elements seen
Fungal cultures	No growth
Meningitis/Encephalitis panel PCR*	Enterovirus detected
Enterovirus PCR	Detected
Cell count/cytospin	WBC 18 (neutrophils 3%, lymphocytes 62%, monocytes/macrophages 32%, eosinophils 0%, basophils 0%, lymphocytes reactive 3%), RBC <1
CSF lactate (1.1 -2.8 mmol/L)	1.3 mmol/L
AFB smear	AFB not seen
AFB cultures	No growth
Respiratory investigations	
SARS-CoV-2 RNA PCR from nasopharyngeal swab	Negative
Respiratory multiplex PCR from nasopharyngeal swab**	Negative
Enterovirus culture from throat swab	Negative
Urine investigations	
Urine CMV DNA PCR	Not detected
Urine formed element	RBC <1 negative, WBC <1 negative, epithelial cells seen, no bacteria seen
Urinalysis	Negative for leucocyte esterase/nitrite/protein/blood
Urine culture	No bacterial growth
Stool investigations	
Enterovirus culture	Positive
Enterovirus PCR	Enterovirus PCR detected, hEV71 PCR not detected
Sanger sequencing	Sabin-like poliovirus serotype 1 detected. 8 nucleotide changes in VP1 from Sabin 1

* Meningitis and Encephalitis panel PCR contents: Escherichia coli K1, Hemophilus Influenza, Listeria monocytogenes, Neisseria meningitidis, Streptococcus agalactiae, Streptococcus pneumoniae, Cytomegalovirus, Enterovirus, Herpes simplex virus 1, Herpes simplex virus 2, Human herpesvirus 6, Human parechovirus, Varicella zoster virus, Cryptococcus neoformans/gattii.

**Respiratory virus multiplex PCR contents: Influenza A, Influenza B, Respiratory Syncytial Virus, Coronavirus (229E), Coronavirus (OC43), Coronavirus (NL63), Coronavirus (HKU1), Parainfluenza virus Type 1, Parainfluenza virus Type 2, Parainfluenza virus Type 3, Parainfluenza virus Type 4, Metapneumovirus, Enterovirus/Rhinovirus, Adenovirus, Chlamydophila pneumoniae, Mycoplasma pneumoniae, Bordetella parapertussis, Bordetella pertussis, SARS-CoV-2.

When she was transferred to Singapore after 1 month of treatment in Indonesia, she was alert but cried and moved very minimally, with flaccid paralysis of the lower limbs, upper limb athetosis and oral dyskinesia (**Supplementary Video S1**). She was fed through a nasogastric tube. Her skin was mildly lichenified with only very scarce generalized papules, without erythema. She had isolated hepatomegaly and no lymphadenopathy. As differential diagnoses at this point included infective and metabolic causes of encephalopathy, we planned for magnetic resonance imaging (MRI) of the brain and spine, analysis of the cerebral spinal fluid (CSF), and a limited metabolic workup and extensive infectious workup (of blood, respiratory swabs, urine and stool). Differential diagnoses of underlying IEI included SCID with Omenn syndrome, Wiskott Aldrich Syndrome, and hypereosinophilic syndrome secondary to a multitude of possible IEIs. A detailed immunologic evaluation was urgently performed with flow cytometry. This promptly revealed T cell lymphocytopenia (CD3+ cells = 238/μL, CD4+ cells= 111/μL, CD8+ cells= 40/μL) with 99.3% CD45RO+ on CD3+CD4+ cells, absent B cells and normal NK cell counts (**Figure 1**, **Table 1**). The combination of generalized rash, hepatomegaly, eosinophilia and the immunologic picture led to a working diagnosis of T-B-NK+ SCID with Omenn syndrome.

While other investigations were ongoing, a more detailed review of her vaccination records revealed that she had received OPV on day 8 of life. VAPP with polioencephalitis was therefore suspected. CSF investigations returned positive for enterovirus RNA on two PCR-based assays, with pleocytosis (WBC 18 with lymphocytic predominance) (**Table 1**). Sabin-like poliovirus serotype I was subsequently confirmed via Sanger sequencing from a contemporaneously taken stool sample (**Table 1**). Metabolic workup (urine organic acids, plasma acyl carnitine, plasma amino acids, ammonia and lactate) and infectious workup were otherwise negative (**Table 1**). MRI of the brain and spinal cords demonstrated extensive involvement of the motor tracts, with symmetrical T2-weighted hyperintensities in the bilateral thalami and cerebral peduncles, as well as anterior horn cell involvement from T10–T12 (**Figure 2b**). These radiological findings were consistent with the clinical findings of co-occurring paralysis and dyskinesia, and also conveyed an extremely guarded neurological prognosis. The onset of neurological disease at four months of age correlated temporally with the expected nadir of transplacentally acquired maternal anti-polio antibodies.

Genetic testing was pursued to complete the diagnosis of SCID with Omenn syndrome. This identified compound heterozygous pathogenic variants in *RAG1* (paternally inherited c.705dup [p.Leu236Thrfs*6] and maternally inherited c.1675A>T [p.Arg559Trp]), while maternal T-cell engraftment was excluded by short tandem repeat analysis, confirming autosomal recessive RAG1-related Omenn syndrome. In view of the i) extent of irreversible neurological damage, ii) absence of effective antiviral therapy for severe poliovirus infection, iii) well-established dependence of HSCT outcomes upon pre-transplant neurological and infectious status, the family made an informed decision not to proceed with transplantation. Accordingly, the focus of management shifted toward immunosuppression for Omenn syndrome and supportive, comfort-focused care.

In terms of her treatment, intravenous (IV) antibiotics were stopped when bacterial cultures returned negative. She continued to have low grade fevers (37.5-38.5 °C) which were not temporally associated with any changes in rash or neurological status; thus, these were probably in keeping with chronic poliovirus encephalomyelitis. We were concerned about disseminated BCG infection associated with her BCG vaccination, however, since no clinical features were present and CT of her thorax and abdomen was unremarkable, further invasive diagnostic procedures were not pursued. Antimicrobial prophylaxis with trimethoprim–sulfamethoxazole (trimethoprim component 5 mg/kg/day) and itraconazole (at 10 mg/kg/day) were initiated. After transfer back to Indonesia however, the child developed features of BCGitis and was initiated on anti-mycobacterial agents.

In terms of immune dysregulation, IV methylprednisolone was initially tapered off after arrival in Singapore. Two days after complete cessation, however, there was recrudescence of generalized erythroderma accompanied by a marked rise in peripheral eosinophil counts (from 0.6 × 10^9^/L to 3.95 × 10^9^/L). Concurrent lymphocyte subset analysis demonstrated expansion of circulating CD3^+^ T cells (>600/µL), all exhibiting a CD45RO^+^ memory phenotype, consistent with relapse of Omenn syndrome necessitating immunosuppression. Decision was made to use oral prednisolone (1mg/kg/day) instead of ciclosporin to minimize the burden of blood sampling for surveillance of trough levels and renal function, with subsequent improvement of rash and eosinophilia. Ciclosporin (5mg/kg/day) was planned as an add-on in case of insufficient immunosuppression and was eventually initiated in Indonesia. Additionally, intravenous immunoglobulin replacement was continued throughout, at approximately 0.4g/kg three- to four-weekly to maintain IgG troughs above 6g/L.

Neurologically, her clinical status remained largely static during admission. She was awake with preserved awareness and was able to briefly fixate and visually track, but exhibited no vocalization, purposeful reaching, or meaningful motor recovery. Dyskinesia persisted, including intermittent tongue thrusting and athetoid movements of the upper limbs, more prominent on the left. She had marked head lag, trunk and complete flaccid paralysis of both lower limbs with areflexia, consistent with the severe established basal ganglia and lower motor neuron (anterior horn cell) injury. She was continued on oral trihexyphenidyl for symptomatic dyskinesia control. Supportive neurorehabilitative care, including regular physiotherapy and occupational therapy, was instituted to optimize comfort, engagement and maintain joint mobility.

After return to Indonesia, she eventually demised from a febrile illness at 8 months of age, likely due to bacterial sepsis that was unresponsive to empirical antibiotics. Comfort care was prioritized, with the child passing away peacefully, surrounded by her family.

## 3 Discussion

Omenn syndrome is a form of leaky SCID characterized by the expansion of memory T cells of host origin that infiltrate the skin and lymphoid tissues, producing the hallmark generalized erythematous rash, and is often associated with lymphadenopathy and hepatosplenomegaly (16). In Omenn syndrome, a dysplastic thymus retains residual but aberrant function, allowing the escape of some T cell clones to the periphery; together with a loss of central tolerance and the defective generation of Treg cells, there is an uncontrolled Th2 type inflammation, and tissue infiltration of clonally expanded T cell clones (17). Therefore, T cell counts may be falsely normal or elevated; detailed immunophenotyping with CD45RO and CD45RA is necessary to reveal the memory phenotype of these T cells and clinch the diagnosis. While Omenn syndrome is usually caused by hypomorphic mutations in *RAG1* or *RAG2*, expansion of CD45RO+ oligoclonal cells (but of maternal origin) can occur in all cases of SCID and also in transplacental maternal engraftment (TME) (16). As such, flow cytometric evaluation that is restricted to T/B/NK-defining surface markers is insufficient for the diagnosis of SCID. No formal guideline currently defines the minimum number of colors or parameters required in diagnostic flow cytometry for IEIs. However, recent multicenter data demonstrated that using only TBNK-defining markers may miss up to one-fifth of IEI cases, underscoring the need for inclusion of naïve and memory T- and B-cell subsets within diagnostic panels (18). While diagnostic flow cytometry is increasingly available in countries in the Asia-Pacific region, the range of surface markers offered may be heterogeneous (14). In our patient, use of a limited panel reduced accuracy of diagnostic evaluation in the first month of treatment.

The presence of VAPP greatly altered the risk-benefit ratio of undergoing HSCT for this family. VAPP occurs at a rate of 2–4 cases per million births per year in the context of OPV use, the vast majority of these occur in patients with immunodeficiencies (11). Brain involvement in polio (polioencephalitis) occurs more often in IEIs which include a humoral defect, representing a more severe disease with higher mortality and morbidity. In 1988, The World Health Organization advocated OPV exclusively for use in developing countries with the goal of polio eradication, because it 1) induces superior mucosal immunity, 2) is easier to administer and 3) is more affordable than inactivated polio vaccine (IPV) (USD 0.15 per OPV dose versus USD 2.50 per IPV dose) (19, 20). While OPV has saved millions of children from death and paralysis, the risk of VAPP and generation of VDPVs – often from the chronic poliovirus infections in immunocompromised hosts that VAPP represents – actually render OPV incompatible with the ultimate goal of polio eradication. Thus in 2008, this prompted the World Health Assembly to prioritize the replacement of OPV with IPV in developing countries as part of an endgame strategy (20). However, in low- to middle-income countries, sustaining polio immunization in a decentralized health-system has proven difficult due to limited fiscal capacity, logistical gaps, and varying commitment across districts (21). Cost remains the single largest determinant preventing nationwide transition to IPV, compounded by the technical challenge of intramuscular administration and supply-chain limitations (21). As such, VAPP remain concentrated in low- and middle-income countries facing logistical and financial barriers to IPV transition, a burden borne mainly by immunodeficient infants in these countries, but with broader repercussions for the global eradiation of polio (11).

The advent of SCID NBS has drastically changed the landscape of SCID by reducing both age and infection-related complications pre-HSCT (22, 23). Although its benefits are undisputed, financial and technical issues remain major obstacles to implementation in public-health programs worldwide (24). As of 2025, the only country in the Asia-Pacific region where universal SCID NBS is available is Singapore (14). Other than cost, the implementation of SCID NBS must also consider existing vaccination schedules for live vaccines, particularly Bacille Calmette–Guérin (BCG) and OPV which are commonly given within the first month of life. Because complications related to the live BCG vaccine are readily treatable, Singapore and other countries have successfully implemented universal SCID NBS while continuing routine BCG vaccination at birth before SCID screening results are known (7). However, since there are no effective antivirals for polio, SCID results need to be known prior to OPV vaccination, which is another aspect of service coordination required in the implementation of SCID NBS.

It is worth noting that families with higher incomes may be able to access advanced diagnostic and treatment options—often concentrated in better-resourced urban centers—even within low- and middle-income countries. In our case, the patient’s family was able to obtain resource-intensive interventions, including pre-implantation genetic testing (see patient perspective). However, SCID NBS – a population-level intervention which requires investment at the health system level – was not accessible to them. Thus, even when certain therapies are available to those who can pay out of pocket, meaningful and sustained improvements in child health depend on population-level interventions that improve outcomes for all children. This is central to health equity, both within countries and globally.

This case report has several limitations. Clinical inference relied on the limited information and clinical data available from the referring hospital after transfer. In addition, interpretation of immunological parameters was influenced by prior immunosuppressive therapy and intravenous immunoglobulin administered before and during transfer, potentially affecting lymphocyte counts and inflammatory markers. Despite these limitations, the convergence of clinical, immunological, radiological, and virological findings support the diagnostic conclusions and highlight the important gaps in health systems which are relevant to the care of children with IEIs.

## 4 Conclusion

Our patient’s clinical course reflects global disparities in availability of appropriate diagnostic tools for diagnosis of immunodeficiency, the impact of continued use of OPV in middle- and lower-income countries, and difficulties in implementing SCID NBS. The case highlights broader global health inequities and its potential impact on global polio eradication efforts, underscoring the urgent need for strengthening diagnostic capabilities, vaccine policy reforms and universal SCID NBS.

## 5 Parents’ perspective

C was born into a family that had long hoped and prepared for her arrival. As first-time parents, we wanted to give her the best possible start in life. Even before her conception, we underwent extensive genetic testing and chose *in-vitro* fertilization with preimplantation genetic testing for aneuploidy (PGTA) to ensure a healthy embryo. During pregnancy, we continued with detailed prenatal genetic testing and advanced 3D ultrasound scans to confirm that every part of her tiny body was perfectly formed. When she was finally born — crying loudly and appearing completely healthy — we were overjoyed.

We never imagined that all our best intentions would turn into a tragedy. Despite doing everything possible to safeguard her health, C was later diagnosed with Omenn syndrome, a severe immunodeficiency that made her unable to fight infections. Both of us were healthy and had no family history of genetic disease, yet we later learned that we were silent carriers of RAG1 variants. What we thought were acts of protection — giving her routine oral polio (OPV) and BCG vaccines — instead became the source of her devastating illness.

After months of struggle, C passed away peacefully, free from pain. As parents, our grief is profound, but we share her story in the hope that no other child or family will have to experience the same loss. We hope her case will raise awareness of immune disorders like Omenn syndrome and the urgent need for newborn screening for immunodeficiency. No infant with immune deficiency should ever again receive a live vaccine such as OPV — safer alternatives like IPV must be prioritized.

C’s short life has become our reminder that early diagnosis and system-level change can save others.

## Data Availability

The raw data supporting the conclusions of this article will be made available by the authors, without undue reservation.
